# Buffalo Academy of Medicine

**Published:** 1900-05

**Authors:** Thomas F. Dwyer


### Society Proceedings.

### BUFFALO ACADEMY OF MEDICINE.

Reported by THOMAS F. DWYER, M. D., Secretary.

THE regular quarterly meeting of the Buffalo Academy of Medicine was held at the Academy parlors, Palace Arcade, Tuesday, March 20, 1900. The meeting was called to order at 8.45 p.m. by the President, Dr. M. D. Mann.

The minutes of the last quarterly meeting were read and approved.

The nomination of officers resulted as follows: For president, Dr. Marcell Hartwig; Dr. Charles Cary. For treasurer, Dr. Charles S. Jewett. For trustees, Drs. John J. Walsh, J. W. Grosvenor, A. A. Hubbell.

The council recommended the election of Drs. Charles A. Cullinane and Charles R. Borzilleri for resident membership, who were separately balloted for and duly elected Fellows of the Academy. The council reported that they had no recommendation to make in reference to change of meeting place and referred the matter back to the Academy.

It was moved by Dr. H. E. Hayd and duly seconded that the Academy remain in the present rooms and the secretary be empowered to make a new lease.

Dr. A. L. Benedict offered an amendment that incorporated an appreciation of the offer of the faculty of the University of Buffalo.

Dr. Arthur W. Hurd offered a further amendment that a committee consisting of the trustees be appointed to select a meeting place for the Academy. This amendment was carried.

The following Assembly Bill, known as the Week's Bill was then read:

An act to amend chapter 546 of the laws of 1896, known as the state charities law, by adding an additional article thereto, to be known as Article IX.*a*, providing for the establishment and management of a state charitable institution for the detention, treatment, reformation and instruction of persons convicted of habitual vagrancy, drunkenness or disorderly conduct, and of persons diseased, incompetent or dangerous from the habitual use of alcoholic stimulants, opium or other narcotics.

Dr. J. W. Grosvenor moved that the Academy recommend the passage of said bill.

Dr. DeLancey Rochester moved that the motion be laid on the table. Carried.

An adjournment was taken to the University of Buffalo where Dr. H. R. Gaylord gave an interesting talk on the present status of recent investigations on cancer, with demonstrations by stereopticon.

Attendance, 67. Adjourned at 11 p. m.

---

*Section on Obstetrics and Gynecology, February 27, 1900.*

Reported by A. J. COLTON, M. D., Secretary.

A REGULAR meeting of this section was held in the Academy parlors, February 27, 1900. The meeting was called to order by the president, Dr. H. D. Ingraham, at 9 p.m.

By order of the president, the minutes of the previous meeting were not read. As there were no cases to be reported or specimens presented, the essayist of the evening, Dr. Edward J. Ill, of Newark, N. J., by invitation, read a paper entitled,

THE INDICATION FOR AND ELECTION OF THE OPERATION FOR UTERINE MYOMA.

In presenting the subject Dr. Ill said he had not arrived at the conclusion that the mere presence of a myoma was an indication for operation, but regarded the patient's welfare as the proper guide. He then classified cases that present for operation into three groups:

(*a*) Those in whom life is or may be endangered directly or indirectly by the growth.

(*b*) Those in whom health is so impaired that life becomes a burden, though not endangered.

(*c*) Those in whom mentality suffers because of the presence of the tumor.

In group *a* he classed (1) the bleeding myoma; (2) the septic or sphacelated myoma; (3) the pressure producing tumor; (4) the growth that becomes strangulated by torsion or impaction; (5) extensive and recurrent inflammatory diseases of the appendages; (6) myomata in the pregnant uterus; (7) myomata in women under 30 years; (8) doubtful diagnosis; (9) attacks of peritonitis, or inflammation of the tumor; (10) the location of the tumor; (11) suspicion of malignancy; (12) excessive serous, mucus, or mucopurulent discharges; (13) severe uterine contractions at the time of menstruation, causing presentation of the tumor at the os externum and thus likely a septic infection; (14) tumors growing after the menopause; (15) inversion of the uterus due to myomata.

In group *b*, he classed (1) large tumors of size sufficient to prevent proper exercise; (2) tumors of excessive mobility; (3) pedunculated tumors adherent to mesentery, bowel or bladder; (4) inflammatory condition of the appendages, causing pain and uneasiness; (5) excessive menstrual pain where the flow becomes obstructed; (6) pain in general.

In group *c* he classed (1) those sensitive women, usually unmarried, in middle life with growths large enough to cause unpleasant comment; (2) women whose mental conditions are such that they brood over their condition, and border on melancholia; (3) women who cannot mentally disassociate the idea of a tumor from cancer.

Dr. Ill took up each of these groups and classes, discussed each in detail, to a greater or less extent, according to its importance, and set forth his views clearly and forcefully. He then presented the contraindications for operation as follows: (1) when the patient is unaware of having a growth or any symptoms therefrom; (2) when she discovers the growth but suffers neither mentally nor physically; (3) when the symptoms are of minor importance; (4) when she can nurse herself, suffers little, and can afford to be carefully watched; (5) when she has passed the menopause, having an old, and likely calcareous, tumor causing few if any symptoms; (6) when near the menopause and can remain under observation until it is passed; (7) when suffering from other and more serious disease likely to lead to fatal results.

Dr. Ill next proceeded to discuss the choice of operation. He did not favor so-called palliative operations, and affirmed that removal of tubes and ovaries with a view to avert growth could be called neither palliative nor conservative. He also declared himself out of sympathy with ligation of the uterine arteries, the galvanic current, and the like, and said that the curette, though admissible, was a dangerous instrument. He favored the conservation of the uterus and said that normally functionating genitalia should be conserved if possible, and affirmed that the conditional point of the conservative operation should be slight mutilation of the uterus and the very complete removal of the tumor.

The author next considered in detail conservative and nonconservative operations, speaking of the vaginal and abdominal routes, and assigning to each its proper place. By each a conservative operation could be made, and by each, as well, a nonconservative. By a combination, too, an abdomino-vaginal operation could be made. He said the vaginal route should be chosen (1) when it has been decided that conservation of the uterus is impossible; (2) when the tumor is freely movable above the linea terminalis; (3) when the vagina is wide enough for easy and comfortable access; (4) when there is complete chronic inversion of the uterus with myomata.

By the abdominal route, he said, we have choice between (1) total extirpation, and (2) supravaginal amputation. He is not partial to total extirpation, though it is indicated where malignancy is suspected, and in some other conditions. He prefers supravaginal amputation with dropping of the stump, when conditions favor this method. He said, that surgeon would prove most successful and receive the largest amount of satisfaction from his work, who strictly followed surgical principles.

DISCUSSION.

Dr. H. E. Hayd said in substance that it was the best paper on the subject he had ever heard and complimented Dr. Ill very highly on his classification of myomata. He said that some operators would remove any and all kinds of uterine tumors and growths no matter how insignificant in size or how unimportant symptomatologically. He praised the writer's technique and was glad to hear him attack the so-called conservative operation of tying the uterine arteries to arrest the growth. It also pleased him to know that Dr. Ill performed the supravaginal operation. In closing, he thanked the writer and reiterated that he had never heard a paper that delighted him so much.

Dr. C. C. Frederick congratulated the writer on his paper and said that he quite agreed with Dr. Ill, that all cases of myomata were not for operation. He said that those myomata not producing any unfavorable symptoms should not be removed. One case, however, he recalled where he advised against operation and afterward regretted it, the tumor becoming inoperable. This he said would not have occurred had the patient been kept under observation. Such cases need careful watching to determine when to operate. No patients upon whom he had operated had suffered return of the tumors. He advised dilating the cervix after supravaginal operations, and in closing complimented and thanked Dr. Ill for coming to Buffalo to read this paper.

Dr. S. Y. Howell in his remarks said the paper in itself was so complete that little was left to be discussed. He paid compliment to the essayist on his scholarly and most admirable paper and the very pleasing manner in which the subject had been presented. He said that it was eminently proper that a vote of thanks be extended to Dr. Ill, which he moved. The motion was seconded and carried.

Dr. CHARLES E. CONGDON said he did not believe in threshing over old straw and so would make no remarks on the points that had been touched upon. He, however, had seen cases of twisted pedicle wherein operation had been refused and which subsequently proceded to fatal issue. There could be no doubt about the propriety of operating in such complications, though diagnosis might be obscure.

Dr. J. W. GROSVENOR asked if there was not a certain class of submucous tumors which were not benefited by the curette. He reported one case in his observation cured by this means.

Dr. W. W. POTTER said it was somewhat difficult to discuss a paper which so completely discusses itself. He said it would no longer do to suggest operation simply because a tumor had been diagnosticated as a fibroid; it must be properly classified as to operable or nonoperable and placed in its proper group according to Dr. Ill's tables. He said the word tumor to some women sounded as badly as cancer does to others, hence one must be careful in the use of the word. He said the exposition of myomatous tumors as presented was the best that he had ever heard; that he particularly felt interested in the portion of the paper dealing with pregnancy complicated by these growths and wished to join with the other members in expressing his personal thanks to Dr. Ill.

Dr. H. D. INGRAHAM said the paper was so clear in its statements and the author so frank and honest in his convictions that it would be difficult to disagree with him. Every one, he said, believes that what the essayist has said is true.

Dr. ILL, in closing, thanked the members of the Academy for the very kind reception of his paper. In answer to Dr. Grosvenor he said that the uterus was apt to become septic after curettement. As to pregnancy mortality after operation, his statistics were that of the gycecologist rather than the general practitioner. He did not agree with Dr. Frederick as to the dilating cervix for drainage, for in his operation, he sewed up the cervix so completely that there was no chance for bleeding. Every bleeding point must be stopped, hence there must be most careful hemostasis before closure of the incision.

The meeting adjourned at 10.30 p.m. There were present 32 members.

---

*Section on Ophthalmology, Otology, Rhinology and Laryngology, March 12, 1900.*

REPORTED BY RICHARD H. SATTERLEE, M. D., Secretary.

REGULAR meeting of the Section of Ophthalmology, Otology, Rhinology and Laryngology, held March 12, 1900. Meeting called to order at 8.45 p.m. Dr. RENNER in the chair.

Minutes of the last meeting were approved.

Dr. F. W. Hinkel exhibited the following cases: I. Chronic suppuration of middle-ear, treated by Stacke's mastoid operation three weeks since. II. Chronic empyema of nasal accessory sinuses, in which antrum, frontal sinus, ethmoid cells and sphenoidal sinus have been opened. (See p. 746.)

Dr. Lucien Howe then presented for the consideration of the section

### OUT-DOOR MEDICAL RELIEF.

He enumerated the various institutions in which relief was given to out-door patients and referring briefly to the manner in which they were conducted, passed at once to the question as to whether there is any necessity for such out-door relief, saying, "These institutions have always existed since the days of the Hospitaler Knights and apparently they will always continue to exist. Those well-managed, will be an honor to themselves, a credit to the physician and a blessing to the patients, while those that are mismanaged will always encourage pauperism, be a discredit to their officers, and but little if any advantage to the patients."

As to a second question—namely, are the dispensaries of Buffalo open to abuse? it is well to remember what this word "abuse" may mean here:

1. Disadvantages to the patient; that may be in two ways: (*a*) Morally, by the encouragement of pauperism in the giving of charity to the well-to-do or to the rich. (*b*) Physically, by exposing those who attend to the contagious diseases of other patients, to inconvenience of crowded waiting-rooms, etc.

2. There is the disadvantage to the physician. That may also be in two ways: (*a*) By the attendant using his position principally to increase his private practice; (*b*) by injuring other physicians in the cheapening of professional services.

As to division 1*a*, the speaker thought that in the past there had been abuses, but that could not be the case now to any extent if the regulations recently adopted by the State Board of Charities are carried out.

In spite of every precaution, some such abuses of charity by patients have occurred, and probably will occur, but this evil has been much magnified in the public mind.

In regard to the disadvantages to the patient already referred to, under 1*b*—namely, those due to exposure to the contagious diseases or overcrowding in waiting-rooms—while these may be factors of importance in metropolitan dispensaries they are hardly worthy of notice in Buffalo.

The abuses which are mentioned in class 2*a* were discussed at length, the conclusion being that this depended largely upon the character of the institution just as standards of professional ethics would vary in individuals. The tendency is, however, even in self-defense, to avoid such abuses; for example, in one of the oldest dispensaries they have a rule which requires each member of the staff to enter in a book the name of each patient referred to an office and also to specify when it was done and exactly why.

Indeed, if this or any other phase of abuse does exist to any considerable extent it can not long continue. Those institutions which intend to comply with both the letter and the spirit of the new law will keep a sharp lookout on any that might not do so, and if that is not sufficient the visitations of the agent of the board of charities will exert a salutary effect in this, as in other ways.

The question was also raised as to why the Board of Supervisors should annually appropriate a considerable sum to the support of infirmaries. Apart from any question of philanthrophy there was apparently sufficient reason because of ultimate economy. Those suffering from most diseases, either die or recover and return to work. If the poor become blind they remain forever dependent on some one, many of them being charges on the county.

A careful estimate was made of the cost of maintaining one blind person during an average lifetime, including the usual course at the Blind Asylum, the minimum cost for the entire life would be about $8,800. If the institutions here which treat over 5,000 annually, and among them a considerable number of young children, should succeed during a whole year in saving from blindness just one child, the county could have made its appropriations in the totals of about $4,000 dollars and still could have gained $4,000 more by the transaction. In this estimate any gain in saving the vision of adults and consequently lessening the number of these supported more or less completely at county expense is entirely left out of the account. Where there are no such institutions, the percentage of blind is decidedly larger than where they do exist. Not only the fact of the saving, but its amount can be determined.

The following resolutions, offered by Dr. Howe, were adopted:

WHEREAS, The members of this Section of the Academy have learned that certain bills now before the Senate and Assembly deeply concern the credit of the medical profession—namely, Senate Bills No. 434, 509 and 511, and Assembly Bills No. 724, 847 and 846; and

WHEREAS, They understand that the general purpose of these bills is to give more control, and therefore more responsibility to the State Board of Charities; therefore,

*Resolved*, That we desire to express to our representatives in the Senate and Assembly our confidence that if such additional powers were granted to the State Board of Charities, it would be used wisely and judiciously.

*Resolved*, That we believe the welfare of patients and the credit of the medical profession would be safer in the hands of one central power than in the trustees of many different institutions, each making regulations for itself.

*Resolved*, That a copy of these resolutions be sent to our representatives in the Senate and Assembly who are now acting on the respective Judiciary Committees.

The section then adjourned.

---

### *Section on Medicine, March 13, 1900.*

REPORTED BY J. C. CLEMESHA, M. D., Secretary.

THE sixth regular meeting of the Medical Section, Buffalo Academy of Medicine, was held at the Academy parlors, Market Arcade, March 13, 1900.

Meeting called to order at 8.45 p.m. Dr. E. H. LONG in the chair. After the minutes of the last meeting were read and approved, Dr. ROCHESTER reported the case of a dwarf, who entered the Sisters' Hospital complaining of difficulty in breathing. There was no rise of temperature and no physical signs of disease in chest or abdomen. In the hospital he developed an attack of facial erysipelas. The pain in the chest and shortness of breath continued and increased. Later he died, and an autopsy showed a marked pericarditis, a broncho-pneumonia, a marked double nephritis and a bladder half full of pus. At one time only did the urine show trace of albumin. The interest in the case lay in the extensive pathological lesions and the absence of symptoms and physical signs.

Dr. CHARLES CARY reported a case of pleurisy with effusion where there had been three tappings and quarts of fluid withdrawn. Examination of the chest showed extensive flatness. Exploration was attempted five times before fluid was reached. Examination of the fluid showed it loaded with cholesterin. Dr. Park operated, removing sections of four ribs and a mass which proved to be a hydatic cyst. No hooklets or scolices were found and the question was whether a diagnosis could be made of such cysts without their presence.

Dr. E. H. LONG referred to a case of chloroform narcosis in which respiration plainly ceased before the circulation, the child fortunately reviving.

The paper of the evening was read by Dr. REYNOLD W. WILCOX, of New York. The subject,

SENILE BRONCHITIS.

After noting that one-third of the cases of death in the aged were due to senile bronchitis, the question of senile involution was touched upon. Tissue changes in old age were due to three factors (*a*) atrophy of tissues, (*b*) infiltration of tissue, (*c*) abnormal development of connective tissue.

The immobility of the chest in the aged and the change in the position of the lungs in the chest were to be considered among others.

The sequence of bronchorrhea, bronchitis, pneumonia and tuberculosis, their symptoms and diagnosis were dwelt upon, and the essayist noted the difficulty of distinguishing clinically senile pneumonia from tuberculosis.

In the treatment of senile bronchitis, belladonna and the expectorants useful in childhood were of little value. The two drugs of first rank were strychnia and ammonium carbonate. To disinfect sputum preparations of creasote were to be used. Opium was an objectionable drug. Inhalations of eucalyptol were of value.

Massage brings about brilliant results and increases the air capacity of the lungs. A warm dry atmosphere, wool next to the skin, the bowels regulated by vegetable purgatives, are requisite for proper conduct of a case; alcohol should be avoided as also hearty evening meals.

DISCUSSION.

Dr. CARY spoke on one point which he considered had been seriously overlooked, *i. e.*, the eliminative function of the lung in old age He considered the cause of senile bronchitis due to the elimination of morbid products by the lung and consequently a toxic disorder. The lung in the aged takes on the work of broken down kidneys. Consequently in the treatment of such a condition, baths and purgatives were indicated and the getting into action all the eliminative organs, was urgently desired.

Dr. STOCKTON stated that senile bronchitis was due to similar changes going on in the skin, kidneys and digestive apparatus. He agreed with Dr. Cary in his view of the eliminative function of the lung, and overwork as a cause of senile bronchitis. The blood is more toxic in the aged and senile bronchitis is a disease of toxemia. The treatment consists of preserving the energy of the kidneys, the elasticity of the skin, the power of the heart and the contractility of the bloodvessels. The hot air vapor bath was one of the foremost therapeutic measures.

Dr. Allen Jones referred to the great value of the iodides, either potassium, sodium or strontium.

Dr. Rochester took into consideration two forms of senile bronchitis: (*a*) Serous expectoration, or little or no expectoration; (*b*) Mucopurulent expectoration. The general treatment in these two classes of cases the same, but the direct treatment is different. In dry cough (serous form) the iodides are useful, while in the second class the balsams are better with the addition of copaiba inhalations.

Dr. H. R. Hopkins mentioned two classes of cases: (*a*) When excretion of urea is diminished; (*b*) when the skin and bowels did not properly exercise their functions.

As for drugs hydriodic acid is the best means of giving the iodide. Next in value is the iodide of sodium; apocynum increased the kidney function and euonymin was excellent for a tired liver.

Dr. Wilcox replied brieflv and the meeting adjourned at 10.25 p.m. Members present, 60.

